# The S4–S5 Linker Acts as a Signal Integrator for hERG K^+^ Channel Activation and Deactivation Gating

**DOI:** 10.1371/journal.pone.0031640

**Published:** 2012-02-16

**Authors:** Chai Ann Ng, Matthew D. Perry, Peter S. Tan, Adam P. Hill, Philip W. Kuchel, Jamie I. Vandenberg

**Affiliations:** 1 Molecular Cardiology and Biophysics Division, Victor Chang Cardiac Research Institute, Darlinghurst, New South Wales, Australia; 2 School of Molecular Biosciences, University of Sydney, Sydney, New South Wales, Australia; 3 St Vincent's Clinical School, University of New South Wales, New South Wales, Australia; 4 Mechanistic Systems-biology NMR Group, Singapore Bioimaging Consortium, Agency for Science, Technology and Research, Singapore, Singapore; Sackler Medical School, Tel Aviv University, Israel

## Abstract

Human *ether-à-go-go*-related gene (hERG) K^+^ channels have unusual gating kinetics. Characterised by slow activation/deactivation but rapid inactivation/recovery from inactivation, the unique gating kinetics underlie the central role hERG channels play in cardiac repolarisation. The slow activation and deactivation kinetics are regulated in part by the S4–S5 linker, which couples movement of the voltage sensor domain to opening of the activation gate at the distal end of the inner helix of the pore domain. It has also been suggested that cytosolic domains may interact with the S4–S5 linker to regulate activation and deactivation kinetics. Here, we show that the solution structure of a peptide corresponding to the S4–S5 linker of hERG contains an amphipathic helix. The effects of mutations at the majority of residues in the S4–S5 linker of hERG were consistent with the previously identified role in coupling voltage sensor movement to the activation gate. However, mutations to Ser543, Tyr545, Gly546 and Ala548 had more complex phenotypes indicating that these residues are involved in additional interactions. We propose a model in which the S4–S5 linker, in addition to coupling VSD movement to the activation gate, also contributes to interactions that stabilise the closed state and a separate set of interactions that stabilise the open state. The S4–S5 linker therefore acts as a signal integrator and plays a crucial role in the slow deactivation kinetics of the channel.

## Introduction

The human *ether-à-go-go*-related gene (hERG) encodes a voltage-gated K^+^ channel, which passes a major repolarising current (termed *I*_Kr_) [1], [2] during the cardiac action potential [3]. HERG channels have unusual gating kinetics characterised by slow transitions between the open and closed states but much more rapid transitions between the open and inactivated states [4], [5]. These unusual gating kinetics are critical both for the role hERG channels play in normal cardiac repolarisation [3] as well as in suppression of premature beats in the period surrounding the termination of the action potential [5], [6]. Loss of hERG function, due to inherited mutations [1], [7] or drug block (Sanguinetti *et al.*, 1995) significantly increases the risks of life threatening arrhythmias, which underscores the importance of these channels.

In recent years, a broad consensus has emerged as to the mechanisms that regulate activation and deactivation of voltage gated ion channels. Changes in membrane potential result in movement of the voltage-sensing domains (VSD, composed of transmembrane helices S1–S4), which are coupled via the S4–S5 linker to the pore domain (S5 and S6 transmembrane helices) resulting in splaying open of the activation gate at the cytoplasmic end of the pore domain [8]. Studies of hERG K^+^ channels have confirmed that each of these elements; the VSD [9], [10], [11], S4–S5 linker [12], [13] and distal pore domain [14] play important roles in activation and deactivation gating of hERG channels. In addition to these components, deactivation gating is modulated by cytoplasmic domains [15], [16], [17], [18], [19], [20], [21], [22]. Numerous previous studies have investigated the effects of mutations in the hERG S4–S5 linker and identified important roles for Asp540 [12], [13], [23], [24] and Gly546 [17], [23], [25] in regulating deactivation kinetics. There has not, however, been a systematic and comprehensive analysis of the effects of mutations throughout the S4–S5 linker.

Here, we show that the solution structure of a peptide corresponding to the S4–S5 linker of hERG contains an amphipathic helix. An analysis of mutations throughout this region shows that the majority, including at the previously identified residue Asp540, are consistent with a role in coupling VSD movement to the activation gate. However, mutations to Ser543, Tyr545, Gly546 and Ala548 had more complex phenotypes. We suggest that the S4–S5 linker acts as a signal integrator and propose a model whereby it both couples VSD movement to pore opening and closure, as well as providing binding site(s), which include Ser543, Tyr545, Gly546 and Ala548, for other domains that regulate activation and/or deactivation of the channel.

## Materials and Methods

### NMR spectroscopy

A 20-residue peptide (L532 to F551) covering the distal end of S4, and the S4–S5 linker of the hERG K^+^ channel was commercially synthesised (GL Biochem, Shanghai, China). The NMR sample consisted of 2 mM peptide and 100 mM deuterated dodecylphosphocholine (DPC, Cambridge Isotope Laboratories, Andover, MA, USA) dissolved in a final volume of 400 µl H2O/D2O, 90/10 (v/v). The analysis of amide protons in ^1^H-NMR spectra is facilitated by lowering the pH to minimise the exchange of amide with water, with pH values of 3–4 typically used [26]. However as we wished to maintain conditions as close to physiological levels as possible whilst obtaining well resolved spectra, we used a pH of 6.6. All NMR experiments were performed on a Bruker Advance II 800 spectrometer (Bruker, Karlsruhe, Germany). Two-dimensional (2D) total correlation spectroscopy (TOCSY) [27] experiments were performed at 300 K with MLEV spin-lock of 60 or 90 ms. 2D nuclear Overhauser enhancement spectroscopy (NOESY) [28] experiments were performed at 300 K with mixing times of 200 and 300 ms. Water suppression was achieved using a DIPSI-2 pulse sequence [29] in the TOCSY experiments and standard presaturation pulse sequence in the NOESY experiments. All of the NMR spectra were processed using TopSpin 3.0 software (Bruker, Karlsruhe, Germany).

### NMR assignment, structure calculation and homology model generation

**Figure 1:**
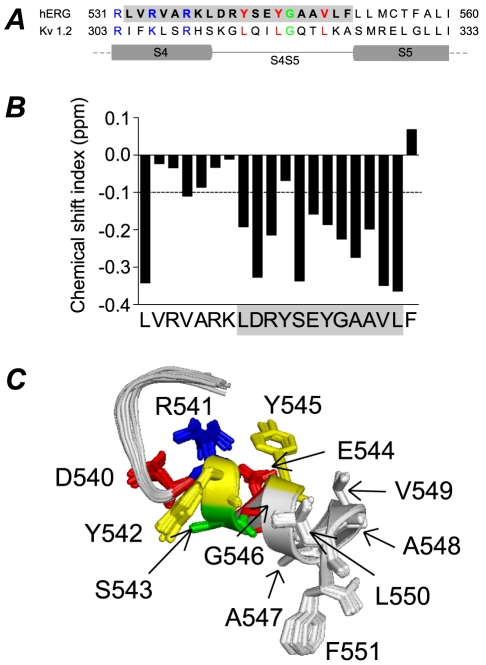
Sequence alignment and structure of hERG S4–S5 linker. A. Sequence alignment of hERG and Kv1.2 for the distal S4, S4–S5 linker and proximal S5 domains. The leucine residues (red) of Kv1.2 S4–S5 linker correspond to tyrosine and valine residues in hERG. Glycine residue (green) in both channels is also conserved. B. Chemical shift index (CSI) plot for NMR structure of hERG S4–S5. CSI values less than −0.1 ppm are indicative of α-helical structure. C. 20 lowest energy structures for hERG S4–S5 with side chains colour coded according to physiochemical properties (basic: blue, acidic: red, polar: green, aromatic: yellow, hydrophobic: grey).

NMR spectra were analysed using the program XEASY3 [30]. Residue assignments were made from 2D TOCSY and NOESY spectra [26]. Automated NOE assignment and structure calculations were performed using the program CYANA 3.0 [31]. Twenty structures with the lowest target function values were energy minimised using the final distance constraints from the CYANA calculation and the generalised Born (GB) implicit solvent model in the program AMBER 11 [32]. The ensemble was validated using the PSVS server [33] and deposited in the PDB [34] under accession code 2LE7. Chemical shift assignments were also deposited in the BioMagResBank under the accession code 17699. A homology model of hERG in the open state was generated using Swiss-pdbViewer and SwissModel [35]. The crystal structure of the open state Kv1.2 K^+^ channel [36] was used as a template. The S4, S4–S5 linker and S5 domains of hERG and Kv1.2 were aligned as shown in Fig. 1A and the S6 domains were aligned using the sequence alignment published previously [37]. The generated homology model of hERG was energy minimised using the generalised Born (GB) implicit solvent model in the program AMBER 11 [32].

### Electrophysiology

HERG cDNA (a gift from Dr Gail Robertson, University of Wisconsin) was subcloned into a pBluescript vector containing the 5′ untranslated region (UTR) and 3′ UTR of the *Xenopus laevis* ß–globin gene (a gift from Dr Robert Vandenberg, University of Sydney). Mutagenesis was carried out using the Quickchange mutagenesis method (Agilent Technologies, CA, USA) and confirmed by DNA sequencing. Wild–type (WT) and mutant channel cDNAs were linearised with BamHI and cRNA transcribed with T7 RNA polymerase using the mMessage mMachine kit (Ambion, Austin, TX, USA).

*Xenopus laevis* oocytes were prepared as previously described [38]. Following isolation, stage V and VI oocytes were stored in tissue culture dishes containing ND96 (in mM: KCl 2, NaCl 96, CaCl_2_ 1.8, MgCl_2_ 1 and HEPES 5) supplemented with 2.5 mM sodium pyruvate, 0.5 mM theophylline and 10 µg mL^−1^ gentamicin, adjusted to pH 7.5 with NaOH and incubated at 18°C. All experiments were approved by the Garvan/St Vincent's Animal Ethics Committee (Approval ID 08/34).

*Xenopus laevis* oocytes were injected with cRNA and incubated at 18°C for 24–48 h prior to electrophysiological recordings. All experiments were undertaken at room temperature (∼22°C). Two–electrode voltage–clamp experiments were performed using a Geneclamp 500B amplifier (Molecular Devices Corp, Sunnyvale, CA, USA). Glass microelectrodes had tip resistances of 0.3–1.0 MΩ when filled with 3 M KCl. Oocytes were perfused with ND96 solution (see above). In voltage dependence of activation and rates of activation protocols, a step of +20 or −30 mV from the holding potential of −90 mV was applied at the start of each sweep to enable off–line leak–current subtraction. We assumed that the current leakage was linear in the voltage range −160 to +40 mV. Data acquisition and analysis were performed using pCLAMP software (Version 10.2, Molecular Devices Corp, Sunnyvale, CA, USA) and Excel software (Microsoft, Seattle, WA, USA). All parameter values were calculated as mean ± standard error of the mean (SEM) for *n* experiments, where *n* denotes the number of different oocytes studied for each construct.

Isochronal activation curves were measured using standard tail current analysis [1]. Cells at a holding potential of −90 mV were subjected to 4–s depolarising steps to voltages in the range of −120 to +50 mV (the precise range depended on the mutant studied) before stepping the voltage to −70 or −120 mV to record tail currents. Tail current data were normalised to the maximum tail current value (*I*_max_) and fitted with a Boltzmann expression:
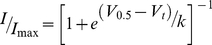
(1)where *I/I_max_* is the relative current, *V*_0.5_ is the half–activation voltage, *V*_t_ is the test potential and *k* is the ‘slope factor’. Alternatively, the data were fitted with the thermodynamic form of the Boltzmann expression:
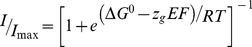
(2)where Δ*G* ^0^ is the work done at 0 mV and *z*_g_ is the effective number of gating charges moving across the membrane electric field, *E*. F is Faraday's constant, R is the universal gas constant and *T* is the absolute temperature. Equations (1) and (2) are equivalent, however, from Equation (2) we can calculate the effect of mutations on changes in chemical potential energy, viz:
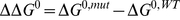
(3)


An envelope of tails protocol was used to measure rates of activation at potentials in the range 0 to +160 mV, as previously described [9]. Rates of deactivation at potentials in the range of −50 to −160 mV were measured as previously described [9]. Rate constants were obtained from fits of a double exponential (or triple exponential function if the recovery from inactivation was also included in the analysis, see Fig. S1) to tail currents recorded at each test potential.

To permit comparison of rates of activation and deactivation in different mutant channels, the observed rates were plotted against *V*−*V*_0.5_, where *V* is the test voltage and *V*_0.5_ is the mid-point of steady-state activation. In previous studies, the total electrochemical driving force, which is a function of both the *V*_0.5_ and k, or slope factor of the steady-state activation curve, see e.g. [9], has been used to correct for shifts in the voltage-dependence of activation and deactivation. We chose not to use this method because the measurement of k from simple Boltzmann fits to steady-state activation data is not particularly robust [39] and because none of the mutations were within the principal voltage sensor region we did not anticipate significant changes to the slope factor. The measured slope factors were in the range 6.9–11.5 mV with the slope for WT channels near the middle at 8.4 mV. For completeness, we have analysed rates of activation and deactivation corrected for driving force, but this makes little difference to any of the relative effects of the mutants compared to WT for rates of activation or deactivation (see Table S4).

## Results

### Solution structure of hERG S4–S5 linker

An alignment of the S4–S5 linker region of hERG and Kv1.2 is shown in Fig. 1A with the peptide sequence used for structural studies shown in bold text. The predicted locations of the C-terminal end of S4 and N-terminal end of S5 are based on the crystal structure of Kv1.2 [36]. In the open/inactivated state crystal structure of Kv1.2 there are three leucines that line one side of the S4–S5 helix and point towards the membrane. These three residues are highly conserved in canonical voltage-gated K^+^ channels (see Table S1). By contrast, in the ether-a-go-go subfamily of voltage-gated K^+^ channels, these residues correspond to Tyr542, Tyr545 and Val549 in hERG, and all are highly conserved within the EAG subfamily (see Table S1). Thus whilst a degree of sidechain hydrophobicity is conserved at these positions, the presence of aromatic sidechains at the first two positions is potentially significant. A centrally located glycine (Gly546 in hERG, Gly318 in Kv1.2) is also conserved. Other residues however, are not well conserved.

The NMR structure of the lower region of the S4 transmembrane helix plus the S4–S5 linker of hERG K^+^ channels (Leu532 to Phe551) was determined in the presence of a deuterated detergent, dodecylphosphocholine (DPC). The NMR chemical shift index plot suggests that the structure contains a coiled region from Val533 to Lys538 and is predominantly α-helical from Leu539 to Leu550 (Fig. 1B). The 20 best NMR structures, superimposed from Asp540 to Leu550 are shown in Fig. 1C. The average backbone and heavy atoms RMSD for all residues are 0.3 and 0.8 Å, respectively. A full summary of the NMR statistics is given in the Table S2.

In Fig. 1C the side chains of Asp540 to Phe551 are shown as stick representations to aid visualisation and the amino acids are colour-coded according to their sidechain properties: red, acidic; blue, basic; green, polar; yellow, aromatic; white, non-polar.

**Figure 2:**
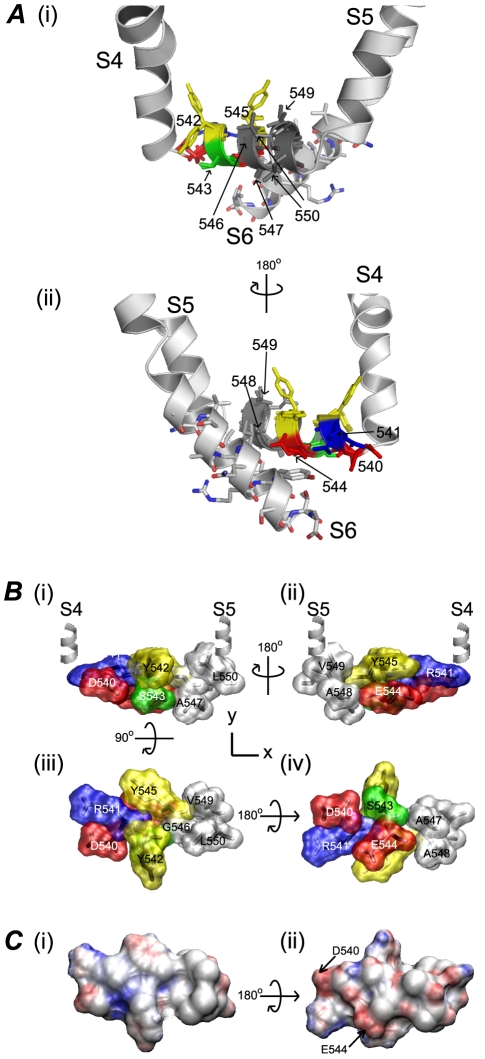
Surfaces of hERG S4–S5 linker. A. NMR structure of hERG S4–S5 linker, from Asp540 to Leu550, superimposed on to the hERG homology model generated using Kv1.2 crystal structure [36] as the template. Only one subunit is shown here for clarity. Each segment is labelled accordingly from S4 to S6. Residues from Asp540–Leue550 are coloured-coded based on their sidechains as in Fig. 1C. View (ii) shows the proximity of the S4–S5 linker to the residues from the S6 in the open state. B. Surface representation of residues Asp540–Phe550. Views shown are: (i) and (ii) S4–S5 linker parallel to the membrane with S4 and S5 helices at either end; (iii) membrane buried and (iv) solvent exposed surfaces. C. The surface electrostatic potential was calculated using APBS software [50]. The membrane-buried surface (i) is neutral whereas the solvent exposed surface (ii) has an overall small negative charge.

Fig. 2A shows the lowest energy NMR structure of the hERG S4–S5 linker superimposed on to an open state homology model generated from a Kv1.2 crystal structure [36]. Apart from the aromatic rings of Tyr542 and Tyr545, which point vertically up in the homology model compared to lying flat in the solution structure, the remaining residues are well correlated. To show the proximity of the S4–S5 linker to the S6 domain, residues from the S6 are also highlighted in Fig. 2A.

Fig. 2B & C show surface representations of the S4–S5 linker from residue Asp540 to Leu550. Panels B(i) and B(ii) show the S4–S5 linker parallel to the membrane with the S4 and S5 transmembrane helices at either end for orientation. Panel B(iii) shows the membrane facing interface including Tyr542 and Tyr545, as well as the bulky hydrophobic residues Val549 and Leu550. By contrast, the surface exposed interface shown in B(iv) is one-half charged or polar residues and one-half non-polar residues. Surface electrostatic potential calculations for the S4–S5 linker illustrate that the membrane-facing surface in the model is neutral, Fig. 2C(i), whereas the solvent exposed surface has an overall negative charge, Fig. 2C(ii).

### Functional role of S4–S5 linker

To investigate the functional role of individual residues in the S4–S5 linker of hERG channels, alanine-scanning mutagenesis was performed on residues Asp540 to Leu550, with the exception of Ala547 and Ala548 which were mutated to valine. Gating parameters including the steady-state voltage dependence of activation, as well as the kinetics of activation and deactivation, were investigated for each mutant channel.

### Steady-state activation

**Figure 3:**
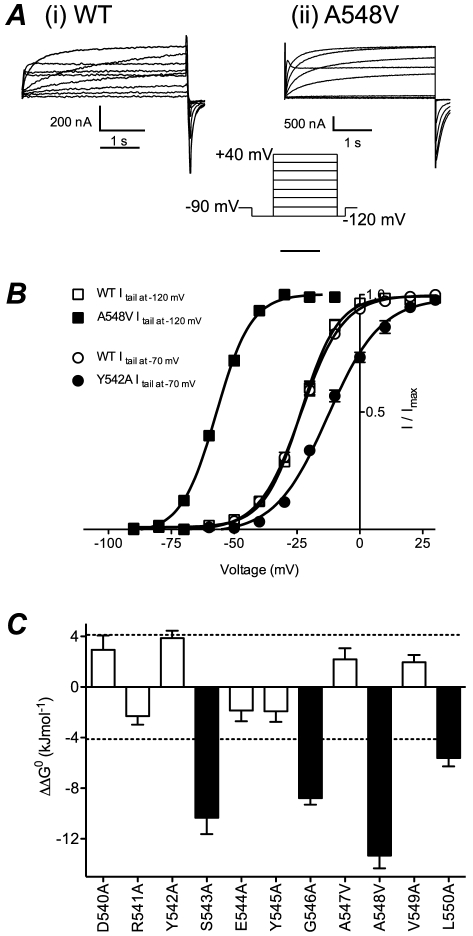
Steady-state activation of hERG S4–S5 linker mutants. A. Typical examples of current traces recorded from (i) WT and (ii) A548V hERG channels using voltage protocol shown in inset. B. Plots of normalised peak tail currents versus test voltage for WT channels, where tail currents were recorded at −120 mV (open squares) or −70 mV (open circles) and for A548V recorded at −120 mV (closed squares) and Y542A recorded at −70 mV (closed circles). In each case the data have been fitted with a Boltzmann function. C. Summary of perturbations to Δ*G* ^0^ of steady-state activation caused by each mutant compared to WT. Filled blocks indicate mutants where ΔΔ*G* ^0^ was >4.2 kJ mol^−1^.

Fig. 3A shows example current traces of steady-state activation for WT and A548V hERG channels. In this instance, tail currents were recorded at −120 mV due to a significant hyperpolarising shift in steady-state activation compared to WT. For mutants that exhibit depolarising shifts in the voltage dependence of activation, such as Y542A, tail currents were recorded at −70 mV (Fig. 3B). To ensure comparisons could be made between mutant channels where tail currents were recorded at different potentials, we show that steady-state activation curves derived from WT hERG channel tail currents recorded at either −70 mV or −120 mV were not significantly different (see Fig. 3B).

There were four mutants that induced significant hyperpolarising shifts in the *V*_0.5_ for steady-state activation: S543A (−48.0±0.8 mV, n = 7), G546A (−63.8±1.2 mV, n = 7), A548V (−56.8±0.5 mV, n = 9) and L550A (−42.0±0.3 mV, n = 6) compared to −23.1±0.8 mV, n = 11 (for WT). The remaining mutants produced only small depolarising (D540A, Y542A, A547V and V549A) or small hyperpolarising (R541A, E544A and Y545A) shifts compared to WT (see Table S3). Fig. 3C summarises the effect of S4–S5 linker mutations on steady-state activation, plotted as changes in chemical potential energy compared to WT hERG channels (ΔΔ*G* ^0^). Mutant channels that induce ΔΔ*G* ^0^ values>4.2 kJ mol^−1^ (equivalent to >1 kCal mol^−1^) are highlighted in black.

### Kinetics of activation

**Figure 4:**
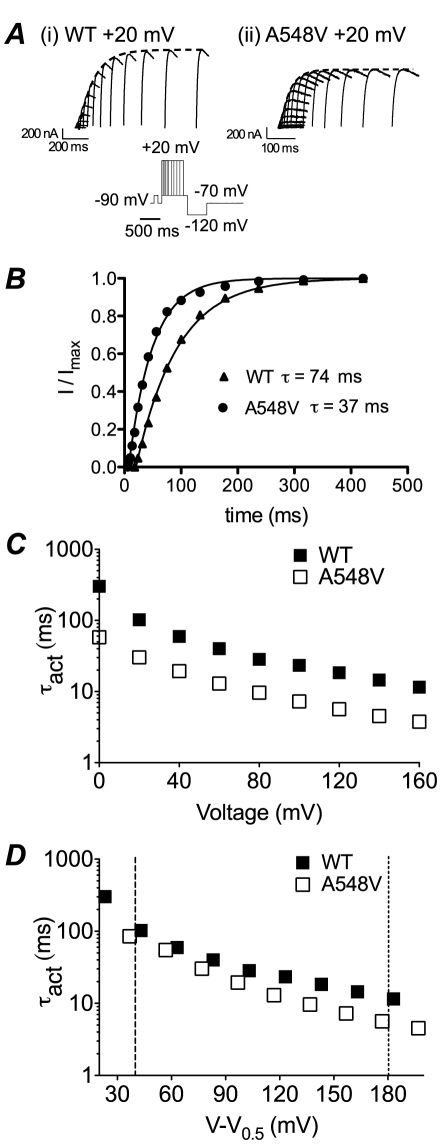
Activation of hERG S4–S5 linker mutants. A. Examples of tail currents recorded at −70 mV from (i) WT and (ii) A548V channels after varying the duration of steps to +20 mV. Dashed lines show single exponential fits to the envelope of peak tail currents. B. Plot of peak tail currents, from experiments shown in panel A, for WT and A548V channels. The fitted single exponential functions had time constants of 74 ms for WT and 37 ms for A548V. C. Plots of time constant of activation for WT (filled squares) and A548V (open squares) channels at voltages between 0 and +160 mV. D. Plots of time constants of activation for WT and A548V versus voltage after correcting for differences in the *V*_0.5_ of activation. Note that the time constants of activation for A548V are no longer markedly different from WT after correcting for differences in the *V*_0.5_ of activation. The dashed lines indicate the voltages (*V* = *V*_0.5_+40 mV and *V* = *V*_0.5_+180 mV) at which comparisons were made for time constants of activation for all mutants.

The rates of activation for S4–S5 linker mutants were measured using an envelope of tails protocol (see methods). Fig. 4A shows typical families of tail currents recorded from (i) WT or (ii) A548V mutant channels. The rate of activation was estimated by fitting an exponential function to the peak of the tail current (Fig. 4A&B). A548V channels activated more rapidly than WT at all voltages studied (Fig. 4C). To determine whether the faster rates of activation for A548V simply reflects the shift in the voltage dependence of steady-state activation, which in turn would create a greater potential gradient driving activation at any given voltage, rates of activation were plotted against *V*−*V*_0.5_ (Fig. 4D). It is clear that after correction for changes in the *V*_0.5_, the rates of activation for A548V are only slightly faster than WT and the difference only becomes significant at the most positive voltages. Similar results were obtained when correcting for electrochemical driving force (see Table S4).

**Figure 5:**
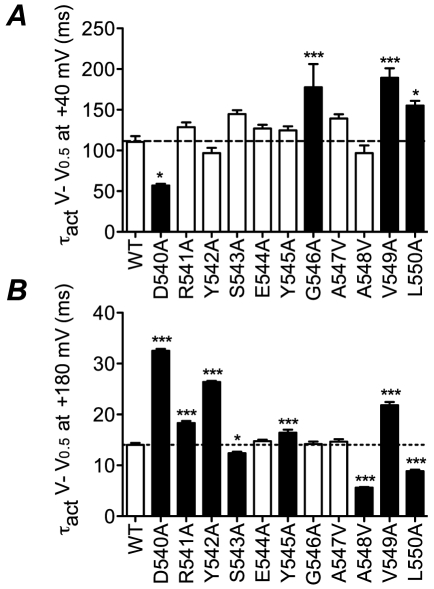
Summary of perturbations to rates of activation. Summary of time constants of activation for all mutants at A, *V* = *V*_0.5_+40 mV and B, *V* = *V*_0.5_+180 mV. Filled bars indicate values that are statistically significantly different to WT (* p<0.05, *** p<0.001).

At voltages close to the *V*_0.5_ of steady-state activation, the last transition to the open state is rate limiting. Conversely, at very high voltages a preceding step, which has low voltage sensitivity, becomes rate limiting [40]. Fig. 5 shows time constants for activation at low voltage gradients (*V*_0.5_+40 mV, panel A) and high voltage gradients (*V*_0.5_+180 mV, panel B) for WT and all mutant channels (see also Table S4). At low voltage gradients D540A activates significantly faster, whereas G546A, V549A and L550A have slower rates of activation and the remainder are not significantly different to WT. Conversely, at high voltages only E544A, G546A and A547V are similar to WT with D540A, R541A, Y542A, Y545A and V549A activating significantly more slowly and S543A, A548V and L550A activating more rapidly than WT. Overall there is no obvious correlation between the effects of the mutants at the two different voltage gradients. This suggests that different residues may contribute to conformational changes underlying the rate limiting transitions at the different voltages.

### Kinetics of deactivation

**Figure 6:**
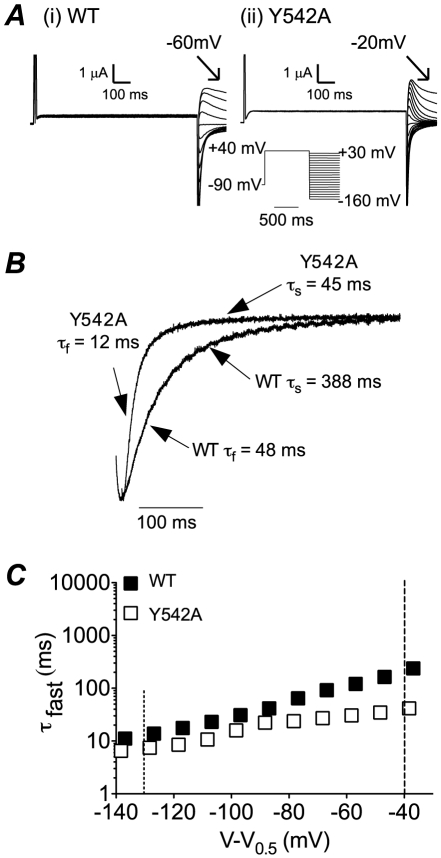
Deactivation of hERG S4–S5 linker mutants. A. Typical examples of families of current traces recorded from (i) WT and (ii) Y542A channels during the voltage protocol shown in the inset. B. Expanded version of tail currents recorded at −110 mV to highlight the much faster deactivation of Y542A compared to WT. C. Plots of time constants of deactivation for WT (filled symbols) and Y542A (open symbols) versus voltage after correcting for differences in the *V*_0.5_ of activation. The dashed lines indicate the voltages (*V* = *V*_0.5_−40 mV and *V* = *V*_0.5_−130 mV) at which comparisons were made for time constants of deactivation for all mutants.

**Figure 7:**
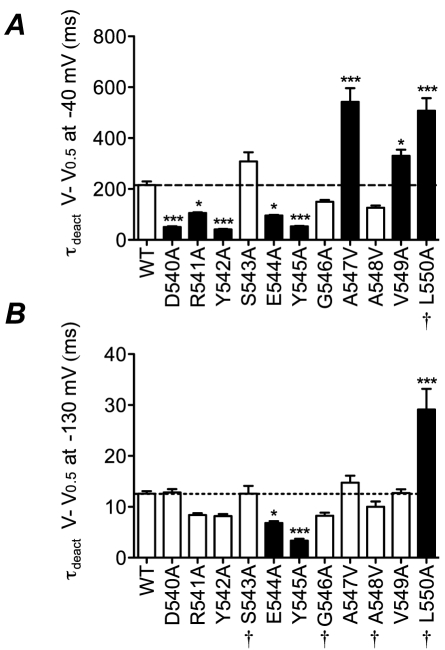
Summary of perturbations to rates of deactivation. Summary of time constants of deactivation for all mutants at *V* = *V*_0.5_−40 mV (A) and at *V* = *V*_0.5_−130 mV (B). Filled bars indicate values that are statistically significantly different to WT (* p<0.05, *** p<0.001). † indicates the mutants where values are estimated values based on extrapolation more than 10 mV from the last measured data point (see Fig. S2).

Typical examples of current families recorded from WT and Y542A channels during a two step voltage protocol to record rates of deactivation are shown in Fig. 6A. Y542A channels have much faster rates of deactivation as can be clearly seen from the normalised expanded tail current trace recorded at −110 mV (Fig. 6B). Deactivation of hERG channels contains a fast and a slow component. However, at potentials less than ∼−120 mV the fast component accounts for well over 90% of deactivation. Accordingly, we have focused our attention on the predominant fast component of deactivation. From Fig. 6C it is clear that Y542A has accelerated rates of deactivation over the entire voltage range tested, after correction for differences in the *V*_0.5_ of steady-state activation (Fig. 6C). The rates of deactivation for all mutants are summarised in Fig. 7 and Table S4. Panel A shows rates of deactivation at low voltage gradients (*V*_0.5_−40 mV) while panel B shows the rates of deactivation at high (*V*_0.5_−130 mV) voltage gradients. It is noteworthy that many more mutants show larger changes in rates of deactivation compared to rates of activation (compare Figs. 7 & 5).

### Overall effect of mutations

**Figure 8:**
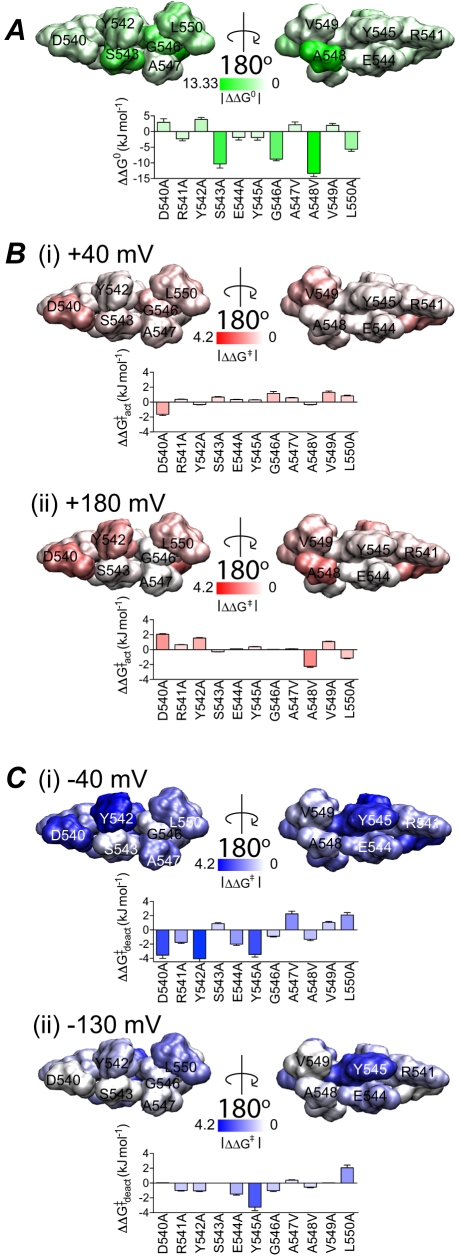
Energy perturbation of S4–S5 linker mutants. The energy perturbation caused by mutations for steady-state activation, rates of activation and deactivation are shown as absolute values and mapped onto the S4–S5 linker viewed parallel to the membrane. The colour scale in steady-state activation A, was normalised to the value corresponding to the largest perturbation (−13.33 kJ mol^−1^) whereas in the case of activation B, and deactivation C, the colour scale spans 0 to 4.2 kJ mol^−1^. B. Perturbations to rates of activation measured at (i) a low voltage gradient: *V* = *V*_0.5_+40 mV and (ii) a high voltage gradient: *V* = *V*_0.5_+180 mV. C. Perturbations to rates of deactivation (fast component) measured at (i) a low voltage gradient: *V* = *V*_0.5_−40 mV and (ii) a high voltage gradient: *V* = *V*_0.5_−130 mV.

To compare the effects of different mutants on changes in the steady-state activation and rates of activation/deactivation, we have mapped the perturbations to the energetics of steady-state activation (Fig. 8A), rates of activation (Fig. 8B) and rates of deactivation (Fig. 8C) onto the structure of the hERG S4–S5 linker. Three of the four mutant channels that show significant hyperpolarising shifts in the voltage dependence of steady-state activation (S543A, G546A, L550A) are located on one side of the helix, with only one mutant (A548V) mapping to the opposite surface (Fig. 8A). The majority of the mutants that perturb kinetics of activation are located on the same surface as Ser543, Gly546 and Leu550 (compare Figs. 8A & 8B). Conversely, the residues where mutations affect rates of deactivation are more widespread (Fig. 8C) and there is no apparent correlation between the effects of mutants on rates of deactivation with steady-state activation.

**Figure 9:**
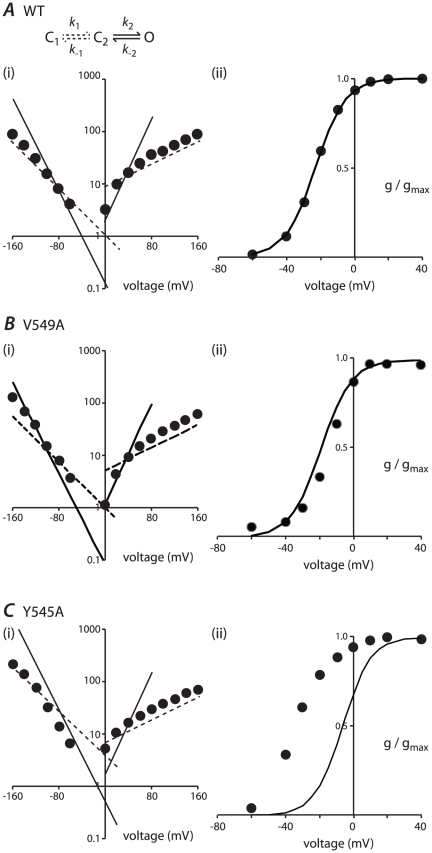
Model based analysis of observed rate constants and steady-state activation. A. Three state transition model, which includes a low voltage sensitivity step (C1 – C2) and a high voltage sensitivity step (C2 – O). The rate constants used in the model fitting are: *k*_1_ = A1(0).exp^0.0125V^; *k*_2_ = A2(0).exp^0.055V^; *k*_−1_ = B1(0).exp^−0.025V^; *k*_−2_ = B2(0).exp^−0.05V^. The values of A1(0), A2(0), B1(0) and B2(0) for WT and all mutants are shown in Table S5. (i) The simulated unidirectional rates of activation/deactivation at low voltage sensitivity (dashed lines) and high voltage sensitivity (solid lines) compared to the observed rates (filled circles) for WT channels. (ii) The modelled steady-state activation (line) closely fits the observed steady-state activation of WT hERG (filled circles). B (i) Çomparison of the simulated rate constants of activation/deactivation (solid and dashed lines) to the observed rates (filled circles) for V549A. Simulated rate constants were obtained by scaling the WT rate parameters using the ratio of the observed rate constants for V549A compared to WT at *V*_0.5_+40 mV (*k*_1_), *V*_0.5_+130 mV (*k*_2_), *V*_0.5_−40 mV (*k*_−1_) and *V*_0.5_−180 mV (*k*_−2_) (see Table S5). (ii) The modelled steady-state activation (line) is similar to the observed state-steady activation (filled circles) of V549A. C. (i) Comparison of the simulated (solid and dashed lines) and observed rates of activation/deactivation (filled circles) for Y545A. (ii) In the case of Y545A, the modelled steady-state activation (line) is very different to that for the observed steady-state activation (filled circles).

If hERG activation was a simple one step process, i.e. C↔O then one would expect that the perturbations to the equilibrium distribution between these two states would be a simple function of the perturbations to the rates of activation and rates of deactivation. hERG activation gating, however, is clearly more complicated and at the very least requires two steps for activation to explain the high voltage-sensitivity of rates of activation at voltages near the *V*_0.5_ of steady-state activation but low voltage sensitivity at very positive voltages. We therefore investigated whether such a two step model (see scheme 1) could explain the data we obtained.
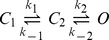
(scheme 1)Arbitrarily, we have designated the first step (C_1_ – C_2_) as having low voltage-sensitivity and the second step (C_2_ – O) as having high voltage sensitivity. We initially investigated whether such a model could reproduce the data for WT hERG. Values for *k*_1_, *k*_−1_, *k*_2_ and *k*_−2_ were systematically varied until they simultaneously reproduced the observed rates of activation and deactivation and reproduced the steady-state activation properties of WT channels (see Fig. 9A and Table S5). Note that with increased magnitude of both positive and negative voltage, the observed rates of activation/deactivation approximate the unidirectional rate constants of the low voltage-sensitivity transition (i.e. *k*_1_ and *k*_−1_). Conversely, at intermediate voltages the observed rate constants more closely follow the unidirectional rate constants of the high voltage sensitivity transition (i.e. *k*_2_ and *k*_−2_). Fig. 9Aii shows that the modelled steady-state activation curve (calculated after a 4 second depolarising pulse using the rate constants from Table S5) closely fits the observed experimental data for WT channels.

We next investigated whether this model could be fit to our data for each of the S4–S5 linker mutants. For each mutant, the rate constants for the low voltage-sensitivity transition (*k*_1_ and *k*_−1_) were scaled by the ratio of the observed rate constants for mutants compared to WT for activation at *V*_0.5_+180 mV (*k*_1_) and deactivation at *V*_0.5_−130 mV (*k*_−1_). The rate constants for the high voltage sensitivity transition (*k*_2_ and *k*_−2_), were scaled by the ratio of the observed rate constants of activation at *V*_0.5_+40 mV (*k*_2_) and deactivation at *V*_0.5_−40 mV (*k*_−2_). We chose to use the rate constants measured at *V*_0.5_±40 mV to correct *k*_2_ and *k*_−2_ since 1) These voltages are sufficiently far from the equilibrium voltage that the observed rate constants approximate the uni-directional rate constants for the transition and 2) At these voltages the rates were sufficiently slow, compared to the rates for the voltage insensitive step, that the *k*_1_/*k*_−1_ transitions would have relatively little influence on the observed rate constants. Using these modified rate constants, the model was used to predict the *V*_0.5_ of steady-state activation measured after 4 s depolarisation steps for each of the S4–S5 mutants. The model predicted values were then compared with the experimentally measured *V*_0.5_ for activation.

Fig. 9B shows the modelled and experimental data for V549A. For this mutant, our simple correction of the rate constants was able to closely reproduce the observed effects on steady-state activation (Fig. 9Bii). Similarly the data for D540A and Y542A can be well approximated by our simple two-state model (data not shown). In contrast to this, the same approach resulted in a poor reproduction of steady-state activation for R541A, E544A, A547V, and L550A (differences between the modelled and measured *V*_0.5_ of steady-state activation were in the range of 10–20 mV) and very poor for S543A, Y545A, G546A and A548V (errors≥25 mV).

In the above models, we used correction factors based on rates measured at *V*_0.5_+40 mV, +180 mV, −40 mV and −130 mV. Very similar results were obtained when we corrected the observed rate constants for total electrochemical driving force and then modified the model rate constants using rates measured at +45 kJ mol^−1^ and −35 kJ mol^−1^ (*k*_1_, *k*_−1_) or ±15 kJ mol^−1^ (*k*_2_, *k*_−2_, see Table S6).

## Discussion

### Structure of the S4–S5 linker

The structure we have determined for a peptide corresponding to the S4–S5 linker of the hERG channel is consistent with that predicted from crystal structures of distantly related Kv channels [36], [41] as well as the predicted structure based on chemical shift index measurements that we previously presented for an extended peptide region spanning the majority of the S4–S5 linker and the entire S5 transmembrane helix [42]. The structure we determined shows a longer helical region than that nominally shown by Gayen and colleagues, who found that only a single helical turn satisfied all the structural criteria to be defined as a helix in the shorter peptide (Leu539-Ala548) [43]. However, the structure of the shorter peptide still had a helix-like conformation with all residues having negative chemical shift index values, although in the case of Asp540 and Ala547 the shifts were quite small. Nevertheless, we cannot exclude the possibility that the S4–S5 linker may be quite dynamic and capable of adopting different conformations in different gating states.

The helix in our structure is amphipathic and based on its homology with the Kv1.2 S4–S5 linker, it is likely that when the channel is in the open state the hydrophobic surface will face into the membrane interior and the hydrophilic surface face the cytosol. The most notable feature of the hydrophobic surface is the presence of two aromatic residues, Tyr542 and Tyr545. These tyrosine residues are highly conserved in the EAG subfamily but are not conserved at all in the canonical members of the voltage-gated K^+^ channel family (see Table S1). Comparison of the NMR structure of the S4–S5 linker peptide with a Kv1.2 homology model shows the two tyrosine residues may adopt different orientations to those for the corresponding leucines in Kv1.2 and so one might expect them to contribute to differences in the activation-deactivation gating of hERG compared to other Kv channels. This is certainly the case for Tyr542 compared to mutations of the equivalent leucine residue in Shaker, e.g. the L382V mutation results in a >60 mV shift in the *V*_0.5_ of steady-state activation of Shaker [44]. The polar surface contains three charged residues (Asp540, Arg541 and Glu544). With the exception of Asp540, the residues on the polar surface are less well conserved within either the EAG subfamily or the broader voltage-gated K^+^ channel family (see Table S1). Thus it is more likely that perturbations caused by mutations to these residues will be more specific to hERG K^+^ channels.

### S4–S5 linker mutations affect the voltage dependence of activation

When mutated to alanine (or from alanine to valine) the residues in the S4–S5 helix that caused the largest perturbations to steady-state activation were Ser543, Gly546, Ala548 and Leu550. Surprisingly, mutations to the aromatic residues (Tyr542, Tyr545) or charged residues (Asp540, Arg541, Glu544) did not perturb steady-state activation, despite the alanine mutation resulting in either large changes in side chain size or chemical properties. This however, can be taken to indicate that these residues are unlikely to be involved in significant interactions in either the open or closed state. In previous studies, it has been reported that charge reversal mutations of Asp540 and Glu544 result in significant perturbations to activation [13]. This suggests that the local electrostatic environment around the S4–S5 linker is important for activation but it requires a more significant perturbation than that caused by single alanine mutants for it to have a clearly observable effect.

### Effects of S4–S5 linker mutations on activation gating

Very few mutations had large effects on the rate of activation. The largest observed perturbation, with A548V, was a 2.5 fold acceleration at the most depolarised potentials. This mutant however, had only very modest affects on activation at less positive voltages. D540A was also notable for the fact that it resulted in a significant slowing at the most depolarised potentials, but an acceleration of activation at less positive voltages. This suggests that Asp540 may be involved in distinct interactions at different steps in the transition between the closed and open states. However, given that D540A did not have a significant effect on the *V*_0.5_ of steady-state activation, overall our data suggests that Asp540 is most likely involved in interactions stabilising intermediate steps and/or transition states rather than interactions that stabilise the most stable end states.

### Effects of S4S5 mutations on deactivation gating

Seven of the eleven S4–S5 mutants (D540A, R541A, Y542A, E544A, Y545A, A547V and L550A) caused a ≥2-fold change in the rate of deactivation at *V*_0.5_−40 mV (see Fig. 7). That so many residues affect deactivation, from both sides of the helix, suggests that the S4–S5 linker is involved in multiple interactions during the transition between open and closed states. Interestingly, very few mutants caused significant perturbations to the rates of deactivation at very high voltages, with only Y545A and L550A causing a >2-fold change in the rate of deactivation at *V*_0.5_−130 mV. Again these two residues are on opposite sides of the helix (see Fig. 8) suggesting that more than one interaction with other domains of the channel may still be involved in stabilising intermediate states involved in the transitions at very negative voltages.

### A modelling approach to understand the overall effects of mutations on activation and deactivation gating kinetics

The S4–S5 linker is clearly important for coupling the VSD to the activation gate. However, the variable effects of S4–S5 linker mutations on the kinetics and voltage-dependence of steady-state activation, and the widespread effects on deactivation gating, suggest that the role of the S4–S5 linker may be much more than just simply one of electromechanically coupling the VSD and activation gate. We used a kinetic modelling approach to help us categorise these mutations and identify those that may be involved in additional interactions. We chose to use a model composed of two closed states and one open state (see Fig. 9). Although there is good experimental evidence to justify more sophisticated models [40], [45], we started with the simplest model required to describe the vast majority of the experimental data for WT channels. Even if this simplistic model was not be able to reproduce all the mutant data, the model failures would point to where individual residues were affecting processes not included in the model.

Perhaps the most surprising finding was that for three of the mutants, D540A, Y542A and V549A (see Fig. 9B) the observed perturbations to the rates could be incorporated into our simple model and very closely reproduce the observed effects on steady-state activation. We interpret this result as indicating that these mutations have perturbed the coupling between voltage sensor movement and activation gate opening without affecting other processes. The remaining mutants could be divided into those where the model fits were only a poor approximation of the experimentally observed data (errors of 10–20 mV, i.e., R541A, E544A, A547V and L550A) and those where the model fits were very poor (S543A: error 25 mV; Y545A: error 26 mV; G546A: 49 mV and A548V: 36 mV). Given that our modelling approach is relatively crude and we have made a number of approximations including, for example, not taking into account changes in slope factors, we will focus our discussions only on those mutants where the errors were largest and therefore likely to be the most robust. Specifically, we suggest that these four mutations, in addition to any effects they have on electromechanical coupling of the VSD and activation gate, have affected additional interactions that modify the transitions between open and closed states of the channel.

Residue Gly546 has already been identified as a critical residue for regulating activation/deactivation properties of hERG [17], [25] and our results are consistent with these previous studies. Here, we have shown that Ser543, Tyr545 and Ala548 are also important. S543A caused a large perturbation to steady-state activation but only modest changes in kinetics of activation or deactivation, A548V caused significant perturbations to both kinetics and steady-state activation, whilst Y545A was associated with marked changes to kinetics but minimal change to steady-state activation. The three residues are also located on different surfaces of the S4–S5 helix (see Fig. 2). Overall, this suggests that these residues are likely to be interacting with different domains during the activation/deactivation transitions and quite possibly affecting different steps during the transition from the most stable closed state at negative voltages through to the most stable open state at very positive voltages.

There is considerable evidence to suggest that there are multiple additional states required to explain hERG activation gating, see e.g. scheme 2:
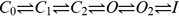
(scheme 2)The third closed state (C_0_) is required to account for the observed sigmoidicity of hERG channel opening [40] and a second open state (O_2_) has been observed in single channel recordings of hERG [46]. How could the perturbations caused by S543A, Y545A and A548V be explained by scheme 2? As S543A causes a marked hyperpolarising shift in the steady-state activation (with relatively little change in kinetics) and removal of the hydroxyl side chain is more likely to destabilise a state than stabilise a state, we suggest that S543A results in destabilisation of a closed state. We cannot however, completely rule out the possibility that replacement of Ser543 with alanine has introduced a new interaction that stabilises an open state. The Y545A mutant had relatively modest effects on rates of activation and steady-state activation but caused a marked acceleration of deactivation throughout the voltage range. This indicates that Tyr545 is most likely involved in interactions that stabilise transition states and/or intermediate steps in the pathway. Given that Y545A perturbed rates of deactivation throughout the voltage range it is also likely that Y545A has destabilised more than one transition state. One caveat with the Y545A mutant, is that removal of the aromatic side chain on the hydrophobic face of the helix could disturb the overall orientation of the helix and so could potentially have more widespread effects than just the local loss of the aromatic sidechain. A548V had more complex effects that suggest destabilisation of multiple states. One caveat with the A548V mutation however is that replacement of alanine with valine will result̀ in an increase in sidechain size and hydrophobicity which could have introduced interactions that stabilise states.

Whilst, our suggestions as to which additional states have been perturbed by S543A, Y545A and A548V are necessarily speculative, it is clear that these mutants must be perturbing states other than those included in our simplified scheme 2 and it is likely to be different states for each mutant. From the literature one can identify multiple potential candidates that could interact with the S4–S5 linker to stabilise closed or open states of the channel. These include the cytoplasmic end of S5 [42], cytoplasmic end of S6 [14], the N-tail [20], [47], the PAS domain [48], a charged cluster in the proximal N-terminus, 362-KIKER-366 [49], and the cNBD in the C-terminus [15]. Identification of the domains that interact with the S4–S5 linker have been an area of significant interest in recent years [24], [47], [48]. Whilst these studies are ongoing, the data provided in this study provides very good clues as to the residues on the S4–S5 linker that are involved in these interactions.

In summary, we have shown that the S4–S5 linker not only couples voltage sensor movement to activation gate opening, but is also involved in interactions that stabilise many of the states along the activation pathway. In addition to Gly546 which has been identified in previous studies, we have shown that Ser543, Tyr545 and Ala548 are likely to be critical residues involved in interactions that stabilise intermediate and/or transition state complexes in the activation/deactivation gating of hERG K^+^ channels. Furthermore, it is likely that each of these residues are involved in stabilising different states and quite possibly are involved in interactions with different domains. Whilst the molecular basis for each of these interactions remains to be confirmed, we suggest that the S4–S5 linker acts as a signal integrator and propose a model whereby it both couples VSD movement to pore opening and closure, as well as providing a binding site for other domains that regulate activation and/or deactivation of the channel.

## Supporting Information

Figure S1 **Fitting of hooked tail current for deactivation of WT hERG at −120 mV.** The tail current corresponding to the voltage step highlighted in dashed box was fitted with either a triple exponential function (red) or biexponential function (blue) to obtain estimates of the fast and slow components of deactivation.
(EPS)

Figure S2 **Extrapolation of rates of deactivation for A548V hERG channels.** Example of extrapolation for value at −130 mV where last measure data point is more than 10 mV from −130 mV. The rates of deactivation were fitted using a single exponential function and extrapolated to −130 mV. In this case, the extrapolated value for tau fast of A548V channel at −130 mV is 8.2 ms.
(EPS)

Table S1 Sequence conservation in S4–S5 linker regions of EAG and Kv channels.
(EPS)

Table S2 Structural statistics of the final 20 ensemble of S4–S5 linker.
(DOC)

Table S3 Activation parameters for mutant hERG channels.
(DOC)

Table S4 Rates of activation and deactivation for mutant hERG channels.
(DOC)

Table S5 Parameters used to simulate forward and reverse rates constants for mutant hERG constructs.
(DOC)

Table S6 Comparison of observed and model-derived values for *V*_0.5_ of steady-state activation.
(DOC)
