# Supplementary material for: The S4–S5 Linker Acts as a Signal Integrator for hERG K+ Channel Activation and Deactivation Gating
Source: PLoS One. 2012 Feb 16;7(2):e31640. doi: 10.1371/journal.pone.0031640 (PMC3280985; doi:10.1371/journal.pone.0031640)
Supplement: Table S2 — Structural statistics of the final 20 ensemble of S4–S5 linker. (DOC) [file pone.0031640.s004.doc]

Table S2. Structural statistics of the final 20 ensemble of S4−S5 linker

| Parameter |  | Ensemble |
| --- | --- | --- |
| Total number of NOE constraints used: |  | 271 |
| Short range | | i – j | < = 1 | 148 |
| Medium range | 1 < | i – j | < 5 | 98 |
| Long range | | i – j | > = 5 | 25 |
|  |  |  |
| Coordinate r.m.s.d. (Å) for all residues |  |  |
| Average backbone RMSD to mean |  | 0.3 |
| Average heavy atom RMSD to mean |  | 0.8 |
|  |  |  |
| Ramachandran assessments |  |  |
| MolProbity (%) |  |  |
| Favored regions |  | 88.1 |
| Allowed regions |  | 11.4 |
| Disallowed regions |  | 0.5 |
