# Supplementary material for: The S4–S5 Linker Acts as a Signal Integrator for hERG K+ Channel Activation and Deactivation Gating
Source: PLoS One. 2012 Feb 16;7(2):e31640. doi: 10.1371/journal.pone.0031640 (PMC3280985; doi:10.1371/journal.pone.0031640)
Supplement: Table S3 — Activation parameters for mutant hERG channels. (DOC) [file pone.0031640.s005.doc]

Table S3. Activation parameters for mutant hERG channels.

| Mutation  (n) | *V*_0.5_  (mV) | *k*  (mV) | ΔG^0^  (kJ mol^-1^) | *z*_g_  (e^-^) | |
| --- | --- | --- | --- | --- | --- |
| WT (11) | –23.1 ± 0.8 | 8.4 ± 0.3 | −6.7 ± 0.3 | | 3.0 |
| D540A (6) | −22.1 ± 1.2 | 11.5 ± 0.7 | −3.8 ± 0.9 | | 2.0 |
| R541A (6) | −31.6 ± 0.9 | 8.7 ± 0.4 | −9.0 ± 0.5 | | 3.0 |
| Y542A (8) | −11.7 ± 1.1 | 11.1 ± 0.6 | −2.8 ± 0.3 | | 2.3 |
| S543A (7) | −48.0 ± 0.8 | 6.8 ± 0.3 | −17.0 ± 1.0 | | 3.6 |
| E544A (9) | −30.1 ± 0.7 | 8.4 ± 0.5 | −8.6 ± 0.6 | | 2.8 |
| Y545A (9) | −32.9 ± 1.2 | 9.5 ± 0.3 | −8.6 ± 0.6 | | 2.7 |
| G546A (7) | −63.8 ± 1.2 | 10.2 ± 0.4 | −15.5 ± 0.2 | | 2.5 |
| A547V (8) | −17.0 ± 0.5 | 7.5 ± 0.1 | −5.1 ± 0.1 | | 2.8 |
| A548V (9) | −56.8 ± 0.5 | 6.9 ± 0.3 | −20.0± 0.7 | | 3.6 |
| V549A (7) | −15.1 ± 0.8 | 7.4 ± 0.2 | −4.7 ± 0.3 | | 3.0 |
| L550A (6) | −42.0 ± 0.3 | 8.2 ± 0.3 | −12.3 ± 0.4 | | 3.0 |
