# Supplementary material for: The S4–S5 Linker Acts as a Signal Integrator for hERG K+ Channel Activation and Deactivation Gating
Source: PLoS One. 2012 Feb 16;7(2):e31640. doi: 10.1371/journal.pone.0031640 (PMC3280985; doi:10.1371/journal.pone.0031640)
Supplement: Table S4 — Rates of activation and deactivation for mutant hERG channels. (DOC) [file pone.0031640.s006.doc]

Table S4. Rates of activation and deactivation for mutant hERG channels.

A) Rates of activation

| Mutation (n)  Rates of Activation | τ act  +40 mV (ms) | τ act  +180 mV (ms) | τ act  +15 kJ mol^-1^  (ms) | τ act  +45 kJ mol^-1^  (ms) |
| --- | --- | --- | --- | --- |
| WT (8) | 110.5 ± 7.5 | 14.0 ± 0.4 | 96.1 ± 4.5 | 17.8 ± 0.2 |
| D540A (5) | 57.0 ± 2.0 | 32.5 ± 0.3 | 38.2 ± 0.7 | 32.4 ± 0.4 |
| R541A (5) | 128.7 ± 5.9 | 18.3 ± 0.4 | 88.6 ± 3.3 | 19.3 ± 0.4 |
| Y542A (6) | 96.9 ± 6.3 | 26.4 ± 0.2 | 49.9 ± 1.3 | 26.3 ± 0.2 |
| S543A (7) | 144.7 ± 4.9 | 12.4 ± 0.3 | 135.9 ± 4.5 | 19.4 ± 0.4 |
| E544A (6) | 127.0 ± 4.5 | 14.7 ± 0.3 | 88.3 ± 2.0 | 15.9 ± 0.3 |
| Y545A (6) | 124.8 ± 4.9 | 16.4 ± 0.6 | 87.4 ± 3.1 | 18.9 ± 0.9 |
| G546A (5) | 177.5 ± 28.7 | 14.2 ± 0.5 | 110.4 ± 13.4 | 13.1 ± 0.5 |
| A547V (6) | 139.3 ± 5.2 | 14.7 ± 0.5 | 84.0 ± 2.8 | 15.0 ± 0.5 |
| A548V (7) | 96.8 ± 9.3 | 5.6 ± 0.2 | 89.5 ± 8.5 | 9.8 ± 0.5 |
| V549A (7) | 189.4 ± 11.6 | 21.8 ± 0.6 | 132.7 ± 6.6 | 23.2 ± 0.6 |
| L550A (5) | 155.0 ± 5.9 | 8.8 ± 0.3 | 93.6 ± 2.7 | 9.3 ± 0.2 |

B) Rates of deactivation

| Mutation (n)  Rates of Deactivation | τ deact (fast)  −40 mV (ms) | τ deact (fast)  −130 mV (ms) | τ deact (fast)  −15 kJmol^‑1^ (ms) | deact (fast)  −35 kJmol^‑1^ (ms) |
| --- | --- | --- | --- | --- |
| WT (9) | 215 ± 14 | 12.6 ± 0.5 | 139 ± 6 | 15.5 ± 0.6 |
| D540A (9) | 50 ± 3 | 12.8 ± 0.7 | 32 ± 1 | 7.9 ± 0.5 |
| R541A (6) | 106 ± 3 | 8.4 ± 0.4 | 74 ± 1 | 10.2 ± 0.4 |
| Y542A (9) | 41 ± 2 | 8.2 ± 0.4 | 25 ± 1 | 5.1 ± 0.3 |
| S543A (8) | 308 ± 34 | 12.6 ± 1.4 | 301 ± 40 | 30.4 ± 2.1 |
| E544A (9) | 95 ± 3 | 6.8 ± 0.4 | 75 ± 3 | 8.2 ± 0.3 |
| Y545A (8) | 53 ± 2 | 3.3 ± 0.4 | 39 ± 1 | 3.6 ± 0.4 |
| G546A (9) | 150 ± 7 | 8.2 ± 0.6 | 205 ± 12 | 10.0 ± 0.4 |
| A547V (9) | 543 ± 54 | 14.7 ± 1.4 | 224 ± 13 | 13.7 ± 1.4 |
| A548V (9) | 126 ± 10 | 10.0 ± 1.2 | 115 ± 8 | 21.2 ± 2.4 |
| V549A (9) | 330 ± 25 | 12.7 ± 0.8 | 193 ± 9 | 15.5 ± 0.9 |
| L550A (6) | 507 ± 49 | 29.1 ± 4.1 | 357 ± 22 | 35.0 ± 4.2 |
