# Supplementary material for: The S4–S5 Linker Acts as a Signal Integrator for hERG K+ Channel Activation and Deactivation Gating
Source: PLoS One. 2012 Feb 16;7(2):e31640. doi: 10.1371/journal.pone.0031640 (PMC3280985; doi:10.1371/journal.pone.0031640)
Supplement: Table S5 — Parameters used to simulate forward and reverse rates constants for mutant hERG constructs. (DOC) [file pone.0031640.s007.doc]

Table S5. Parameters used to simulate forward and reverse rates constants for mutant hERG constructs.

| Mutation | *k*_1_^*^  A1(0) | *k*_-1_^*^  B1(0) | *k*_2_^*^  A2(0) | *k*_-2_^*^  B2(0) |
| --- | --- | --- | --- | --- |
| WT | 8.0000 | 1.0000 | 2.0000 | 0.1250 |
| D540A | 3.4462 | 0.9844 | 3.8772 | 0.5347 |
| R541A | 6.1202 | 1.5000 | 1.7172 | 0.2552 |
| Y542A | 4.2424 | 1.5366 | 2.2807 | 0.6525 |
| S543A | 9.0323 | 1.0000 | 1.5273 | 0.0874 |
| E544A | 7.6190 | 1.8529 | 1.7402 | 0.2825 |
| Y545A | 6.8293 | 3.8182 | 1.7708 | 0.5095 |
| G546A | 7.8873 | 1.5366 | 1.2451 | 0.1798 |
| A547V | 7.6190 | 0.8571 | 1.5865 | 0.0497 |
| A548V | 20.0000 | 1.2600 | 2.2831 | 0.2135 |
| V549A | 5.1376 | 0.9921 | 1.1668 | 0.0816 |
| L550A | 12.7273 | 0.4330 | 1.4258 | 0.0531 |

* *k*_1_ = A1(0) exp^0.0125.V^

*k*_-1_ = B1(0) exp^-0.025.V^

*k*_2_ = A2(0) exp^0.055.V^

*k*_-2_ = B2(0) exp^-0.05.V^
