# Supplementary material for: The S4–S5 Linker Acts as a Signal Integrator for hERG K+ Channel Activation and Deactivation Gating
Source: PLoS One. 2012 Feb 16;7(2):e31640. doi: 10.1371/journal.pone.0031640 (PMC3280985; doi:10.1371/journal.pone.0031640)
Supplement: Table S6 — Comparison of observed and model-derived values for V 0.5 of steady-state activation. (DOC) [file pone.0031640.s008.doc]

Table S6. Comparison of observed and model-derived values for *V*_0.5_ of steady-state activation.

| Mutation | Measured *V*_0.5_ | Modelled *V*_0.5_  (*V*_0.5_ – *V*) | Difference | Modelled *V*_0.5_  (Driving force) | Difference |
| --- | --- | --- | --- | --- | --- |
| WT | -23.1 | -23.1 | 0 | -23.1 | 0 |
| D540A | -22.1 | -14.9 | 7 | -15.5 | 7 |
| R541A | -31.6 | -14.4 | 17 | -17.6 | 14 |
| Y542A | -11.7 | -8.0 | 4 | -10.3 | 1 |
| S543A * | -48 | -23.3 | 25 | -25.7 | 22 |
| E544A | -30.1 | -13.7 | 16 | -17.7 | 12 |
| Y545A * | -32.9 | -6.5 | 26 | -9.4 | 23 |
| G546A * | -63.8 | -14.8 | 49 | -24.2 | 40 |
| A547V | -17 | -26.9 | -10 | -28.1 | -11 |
| A548V * | -56.8 | -21.2 | 36 | -23.8 | 33 |
| V549A | -15.1 | -19.1 | -4 | -21.4 | -6 |
| L550A | -42 | -27.6 | 14 | -32.5 | 9 |

* Mutations where the difference in *V*_0.5_ between model and experiment was >25 mV (for *V*_0.5_ – *V* model) and >20 mV for driving force model.
